# Measurements of the Stiffness and Thickness of the Pavement Asphalt Layer Using the Enhanced Resonance Search Method

**DOI:** 10.1155/2014/594797

**Published:** 2014-09-03

**Authors:** Nur Mustakiza Zakaria, Nur Izzi Md. Yusoff, Sentot Hardwiyono, Khairul Anuar Mohd Nayan, Ahmed El-Shafie

**Affiliations:** ^1^Department of Civil & Structural Engineering, Universiti Kebangsaan Malaysia, 43600 Selangor, Malaysia; ^2^Department of Civil Engineering, Universitas Muhammadiyah, Yogyakarta 55183, Indonesia

## Abstract

Enhanced resonance search (ERS) is a nondestructive testing method that has been created to evaluate the quality of a pavement by means of a special instrument called the pavement integrity scanner (PiScanner). This technique can be used to assess the thickness of the road pavement structure and the profile of shear wave velocity by using the principle of surface wave and body wave propagation. In this study, the ERS technique was used to determine the actual thickness of the asphaltic pavement surface layer, while the shear wave velocities obtained were used to determine its dynamic elastic modulus. A total of fifteen locations were identified and the results were then compared with the specifications of the Malaysian PWD, MDD UKM, and IKRAM. It was found that the value of the elastic modulus of materials is between 3929 MPa and 17726 MPa. A comparison of the average thickness of the samples with the design thickness of MDD UKM showed a difference of 20 to 60%. Thickness of the asphalt surface layer followed the specifications of Malaysian PWD and MDD UKM, while some of the values of stiffness obtained are higher than the standard.

## 1. Introduction

Flexible pavement is a composite material that consists of a mixture of aggregate, sand, bitumen, and filler material which provides a road surface with sufficient skid resistance and function to disseminate vehicle load to the subgrade and at the same time has a long life expectancy without the need for frequent maintenance. Pavement is designed with regard to major aspects, namely, the thickness, strength, resistance to surface water, and texture of the surface, to ensure that it can work properly. The objectives of pavement design generally involve the selection of building materials and ensuring that the thickness of each layer is correct to ensure that the flexible pavement layer is able to protect the subgrade from the impact of the traffic load [1].

However, the quality of flexible pavement decreases over time and depends on the quality of the materials used, the environmental conditions, and the traffic load exerted. Increased traffic load on old road infrastructure will result in a decrease in the thickness of the flexible pavement, shortening its lifespan [2]. Therefore, the exact thickness of each layer should also be emphasized in order to avoid damage occurring to the pavement as a result of the traffic load and the environment. The main aspects that need to be considered in road pavement management systems are an assessment of the current stiffness and the predicted pavement strength in the future [3]. The strength of flexible pavement can be determined by the elastic modulus parameter; this parameter is significant in predicting and evaluating the performance of flexible pavement when static and repetitive traffic loads are applied [4].

In general, falling weight deflectometer (FWD) is widely used equipment or tool to identify stiffness of the pavement system. The FWD is an experimental method of nondestructive test (NDT) which imposes an impulse load on the pavement surface through a circular steel plate where the stiffness of pavement layers system was evaluated by measuring maximum dynamic displacements. The FWD data contains environmental data, layer thickness, material response functions, and traffic load information. This testing tool is widely used as it is easily performed with good and efficient results. Meanwhile, ground penetrating radar (GPR) is another tool that can be used for measuring pavement thickness. To capture the pavement layer systems, a survey vehicle was used to place an antenna that received short pulses of electromagnetic energy from the pavement. The series of pulse will form a radar waveform that contains a record of the properties and thickness of the pavement layers system. This tool is accurate and nondestructive technique for evaluation of pavement layers system.

Recently, Joh et al. [5] developed a new tool, known as a pavement integrity scanner (PiScanner), to evaluate the stiffness for pavement layers system and, at the same time, the thickness of the pavement can be identified. In this study, the enhanced resonance search (ERS) method was used to determine the thickness and stiffness of the asphalt surface layer by using PiScanner. The PiScanner based on the ERS method has been tested earlier on rigid pavement structures; however, the applicability of this equipment for flexible pavement has yet to be investigated. Therefore, this study is conducted to identify the effectiveness of the PiScanner on flexible pavement in determining the thickness of the existing pavement system. The stiffness of the flexible pavement also will be determined, based on the theory of elasticity. However, the evaluation of flexible pavement structure will concentrate only on the asphalt surface layer, which is a combination of the wearing and binder courses.

## 2. Enhanced Resonance Search (ERS) Method

The thickness of the asphalt layer was determined using the ERS method. This method is a combination of the SASW and resonance method, whereby SASW determines the shear wave velocity profile and the thickness of the asphalt is determined using a resonance search [5, 6]. Due to the inadequacy of measuring the pavement layer thickness by SASW, a resonance search of the pavement layer is used to ensure that the thickness of the pavement layer is determined accurately. SASW is used to evaluate the strength of the pavement system and concrete structure using surface wave velocity to determine the elastic properties of the material [7]. This method is also able to determine the elastic modulus and thickness of a layered system by fully utilising the advantages of surface waves [8].

An automation algorithm was developed by Joh et al. [5], for which this new algorithm was used to ease vertical profiling of concrete modulus in rigid pavement because conventional computation requires 15 to 30 minutes to compute theoretical modelling of wave propagation. By using this algorithm, approximately 3 to 5 minutes was taken for the analysis time. Two processes, namely, phase velocity calculation and resonance search, were using this automation algorithm. In automated phase velocity calculation, the fundamental wave group is extracted from the surface wave that propagates. Wave group in a Gabor spectrum will be examined for the extraction. While automated resonance search used iterative comparison of field measurements and theoretical model results in order to find accurate thickness, a resonant frequency from the frequency response curve was defined by using a theoretical modelling.

The use of the frequency domain over the time domain and the wave number of the *R* wave transform analysis has several advantages, including the ease with which the solution to the wave propagation equation in the available frequency domain and the frequency and wave number analysis are obtained. This is because the analysis in the time domain using numerical integration is more complicated [9]. All information about wave propagation is obtained by the analysis of frequency and wave number [10]. Through frequency and wave number analysis, Nolet and Panza [11] show that the production of a spectrum is very convincing. In addition, the use of the Fourier transform has been developed to analyse the spectrum by numerical methods that can calculate digitally, known as the fast Fourier transform (FFT). This technique is able to measure and analyse dynamics systems in the frequency domain.

Body wave measurement is the measurement of the resonance method based on the variety reflection in bounded media. This method is stable in determining the dominant frequency of multiple wave reflections. One resonance method is the impact echo (IE) method. IE involves exerting an impact on the structure surface over a short period of time to produce low-frequency waves. The generated wave will propagate into the structure and then be reflected if there is a defect inside the structure or external borders [12].

## 3. Determination of Stiffness

According to the theory of wave propagation, the stiffness or the maximum shear modulus of the material under 0.0003% of strain can be determined from the velocity of *S* wave propagation or shear wave [13]:

(1) $$\begin{array}{c} G=\rho V_{S}^{2}. \end{array}$$

Based on the elastic theory proposed by Yoder and Witczak [14], the elastic modulus of material can be described as

(2) $$\begin{array}{c} E=2G\left(1+\mu \right)=2\rho V_{S}^{2}\left(1+\mu \right), \end{array}$$

where *G* is shear modulus, *E* is elastic modulus, *ρ* is density, *V*_*S*_ is *S* wave velocity, and *μ* is Poisson's ratio.

## 4. Methodology

### 4.1. Equipment

#### 4.1.1. PiScan Probe

**Figure 1:**
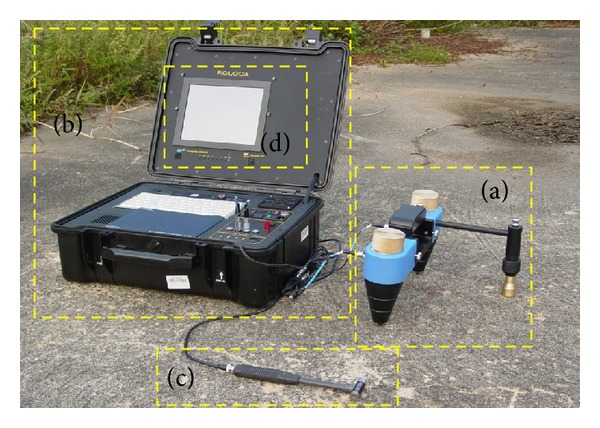
PiScanner system. (a) PiScan Probe, (b) POLCCA, (c) instrumented hammer, and (d) PiScanner Analyzer.

The PiScan Probe (Figure 1(a)) consists of a sensor unit that uses an accelerometer for sensor wave propagation and this wave is created with an instrumented hammer (Figure 1(c)), which functions as an impulse generator. This apparatus is equipped with two accelerometers. The distance between the two accelerometers was adjusted to 0.15 and 0.30 m and there is a weight on each of the accelerometers. This weighting is to ensure that contact between the accelerometer and the pavement surface is perfect [5]. Therefore, any disturbance and the time interval of the detector can be eliminated. Joh et al. [5] explain that although the accelerometer in the PiScan Probe has a special frame, the measured signal is almost equal to the signal measured by the bare accelerometer.

#### 4.1.2. POLCCA

POLCCA (Figure 1(b)) serves as a spectrum analyser that receives wave signals from the PiScan Probe. The wave signal in the form of amplitude and wave propagation time that is detected by the accelerometers will be recorded. POLCCA consists of a PiScan Analyser (Figure 1(d)) which functions as a data analyst equipped with dynamic signal analysis (DSA) or FFT analysis. The analyser performs the ERS measurement and analyses data measured automatically [5]. It is also equipped with a coupling AC/DC, four analogue channels, antidisguise filters, and an analogue trigger.

### 4.2. Set-Up of the ERS Method

**Figure 2:**
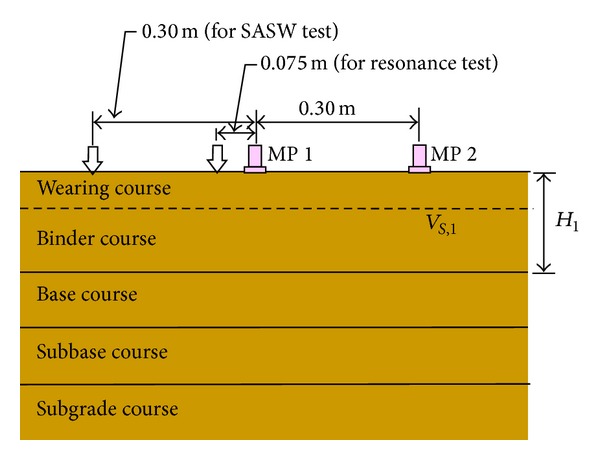
ERS measurement.

The ERS method is a combination of the SASW and resonance methods. Thus, there are two ways to arrange the accelerometers and the impulse generator (Figure 2). For SASW or surface wave measurement, the measurement of the wave signal involves an instrumented hammer and two accelerometers. The distance between MP1 and MP2 is 0.30 m and the distance of the instrumented hammer from MP1 is equal to the distance between MP1 and MP2. The distance between the instrumented hammer and MP1 represents the assumed maximum depth. The resonance method involves an instrumented hammer and an accelerometer where the distance between the hammer and MP1 is 0.075 m.

### 4.3. Site Location

**Figure 3:**
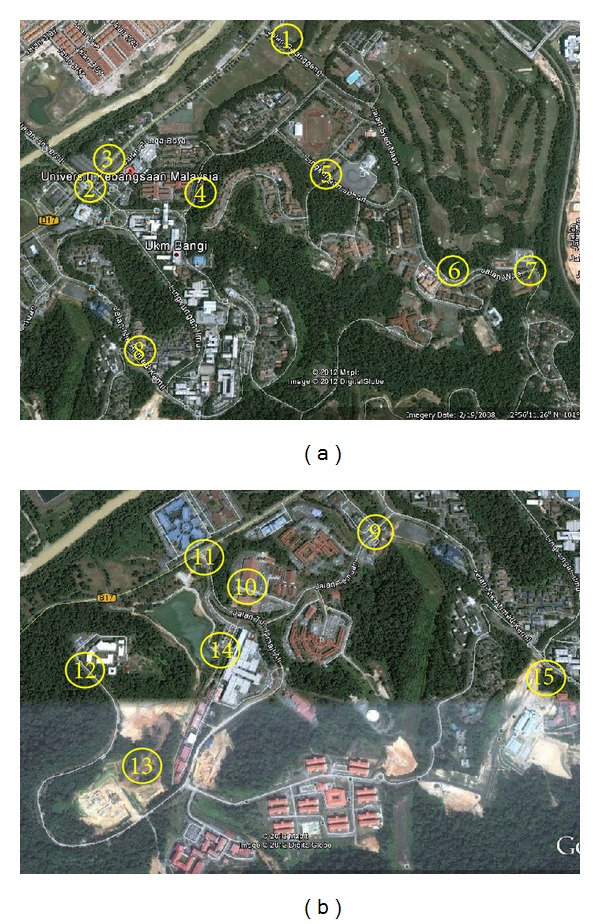
(a) Loop 1 and (b) Loop 2 of UKM campus.

The study focused on the structure of flexible pavement in the main campus of Universiti Kebangsaan Malaysia (UKM), Bangi, Selangor. The study area can be divided into two sections, namely, the first loop and the second loop, shown in Figure 3. A total of eight locations in the first loop and seven locations in the second loop were identified for measurement.

## 5. Analysis and Discussion

### 5.1. Procedure of ERS Analysis

**Figure 4:**
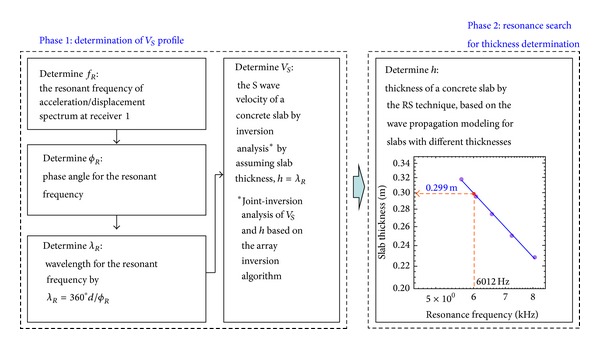
ERS method procedure (Cho et al., 2007 [6]).

In field data analysing, the ERS involves a combination of two methods. Therefore, this method consists of two main phases in the data analysis. The first phase is determining the shear wave velocity profile using SASW; the second phase involves determining the asphalt layer thickness. Surface wave analysis is done in advance to produce a graph of shear wave velocity versus depth so that a more accurate measurement of the asphalt layer thickness can be acquired from body wave analysis.  Figure 4 shows details of the procedures involved.

### 5.2. Seismic Data Acquisition

**Figure 5:**
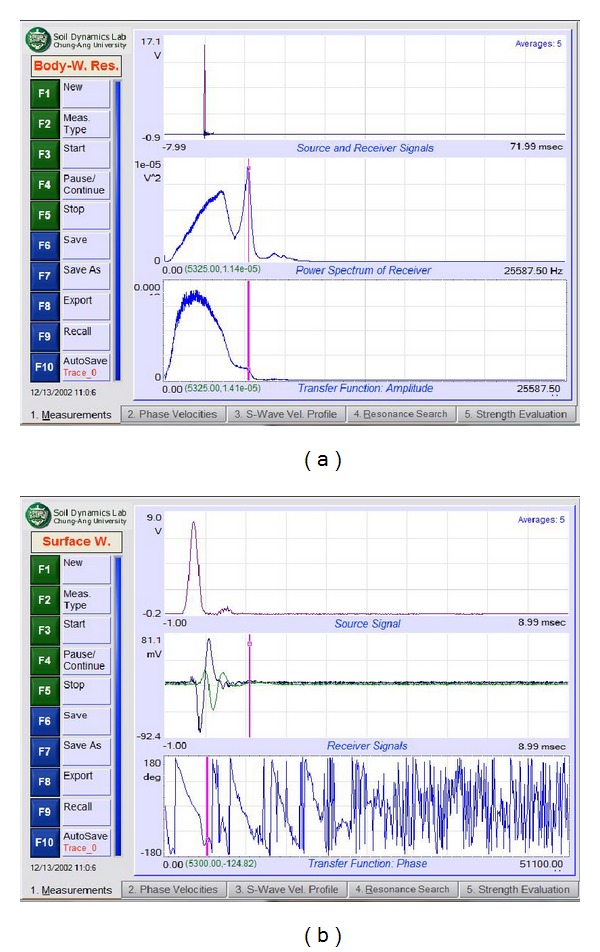
(a) Frequency spectrum of body wave resonance measurement and (b) Phase angle spectrum from surface wave measurement.

There are four main steps involved in seismic data acquisition. The first is the placement of the accelerometer and impulse generator to produce waves. The second step is to set up the PiScanner Analyser to perform the measurement. The third and fourth steps are to measure the resonant body wave and the surface wave. The ERS measurement begins with two accelerometers placed on the pavement surface. These accelerometers should be touching the pavement surface without any holes to ensure that the data obtained are satisfactory. The hammer is then used to create a transient effect on the pavement at two positions, namely, 300 mm and 75 mm from the first accelerometer. The second step is to determine the settings for the PiScanner Analyser. The third step involves collecting body wave resonance signal data with the hammer set at 75 mm from the first accelerometer. Ten hammer blows are applied and the average of the resulting signal is recorded, as shown in Figure 5(a). The final step involved in seismic data acquisition is recording the surface wave. With a distance between the impact source and first receiver set at 300 mm, ten hammer blows are applied and the average of the resulting signal is recorded, as shown in Figure 5(b).

### 5.3. Data Analysis

**Figure 6:**
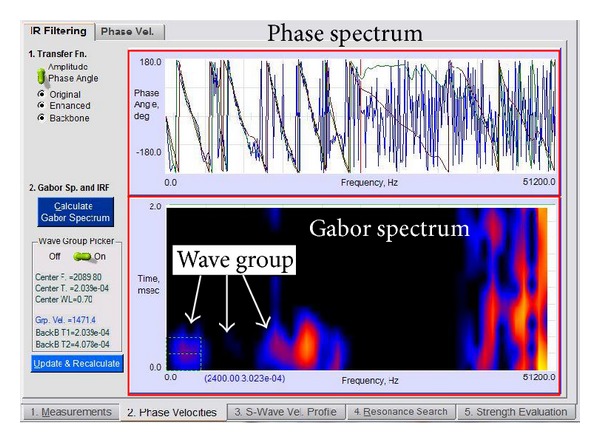
Gabor spectrum (contains wave groups).

Data obtained from the field was analysed using automated algorithm analysis methods. The analysis was divided into two stages: the first stage is the velocity search phase and the second stage involves the resonant frequency search to determine the thickness of the asphalt surface layer. In the phase velocity search, the analysis begins with the production of the Gabor spectrum (Figure 6) of the impulse response. This consists of a group of resulting waves and the frequency of the wave group is used in filtering the pulse response (IR filtering, IRF) to produce a phase spectrum. The Gabor spectrum is the spectrum that displays the concentration of energy that can be produced from the propagation of the wave groups and is used to separate clearly the frequency range of the wave groups propagated at high and low modes. Thus, the arrival of the wave group on the pavement layers and the frequency components belonging to the group of the wave can be determined.

**Figure 7:**
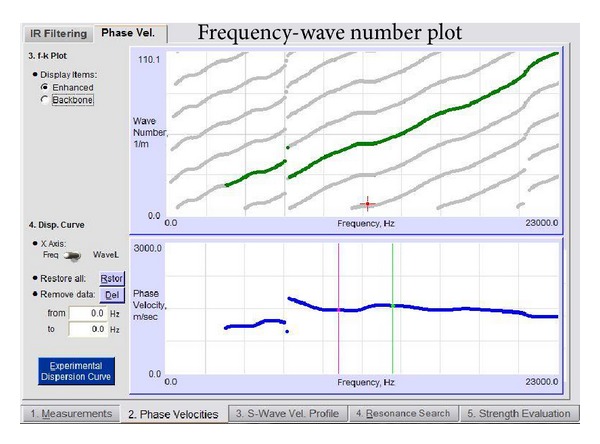
Phase velocity dispersion curve.

**Figure 8:**
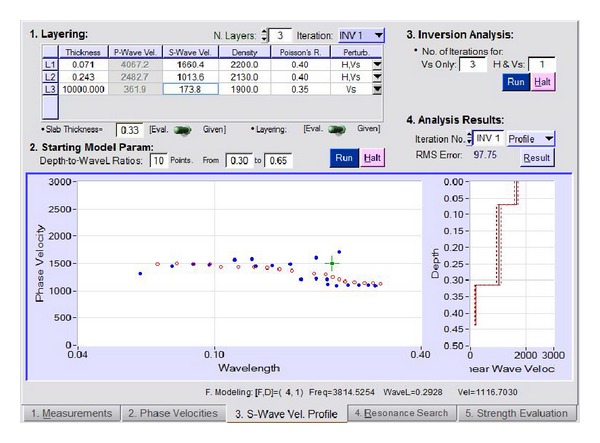
Inversion analysis (determination of the thickness of each pavement layer based on the shear wave velocity (*S* wave)).

The dispersion curves for the phase velocity are then produced from the enhanced spectrum, as shown in Figure 7. The shear wave velocity can be determined from the velocity dispersion curves after going through the inversion analysis. The parameters used in this analysis are shown in Table 1. The data undergoes a layering process and then inverse analysis is done once again to determine the thickness of each pavement layer based on the shear wave velocity (*S* wave) by assuming that the thickness of the pavement layer structure is equal to the resonant wavelength. This analysis uses the phase velocity dispersion curve with the lowest RMS value resulting from the layering process. A comparison of the dispersion curve obtained theoretically and experimentally is depicted in Figure 8.

**Figure 9:**
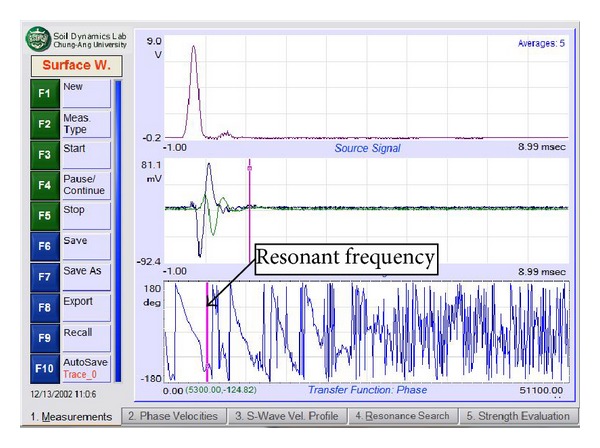
Resonant frequency of phase spectrum at transfer function.

**Figure 10:**
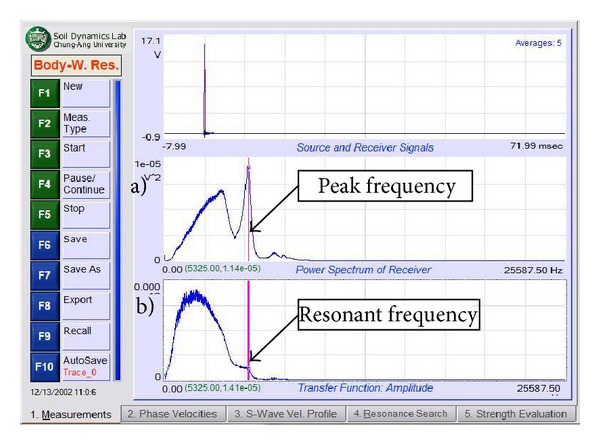
Frequencies generated at (a) power spectrum receiver and (b) transfer function (obtain resonant frequency to determine thickness of asphalt surface layer).

In order to determine the thickness of the pavement more precisely, a second analysis involving the resonant frequency search is done. By default, this search technique is designed to determine the thickness of the pavement layer structure where the thickness of the resonant frequency is equal to the resonant frequency measured. This analysis involves finding the resonant frequency of the phase spectrum of the transfer function that is produced by surface waves (Figure 9). This frequency is used as a reference for finding the peak frequency of the power spectrum at the first receiver, as shown in Figure 10, and then applied to determine the resonant frequency of the body waves produced.

**Figure 11:**
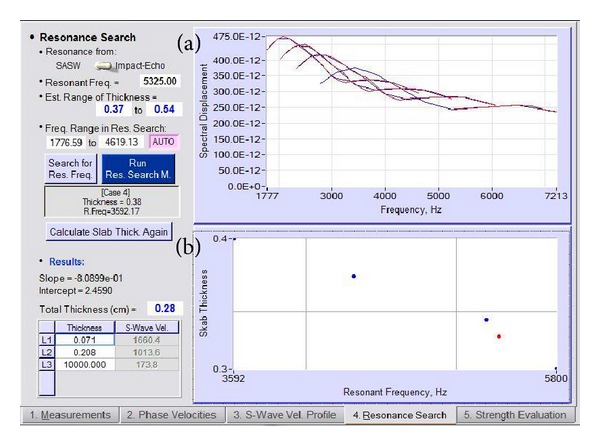
Analysis of resonant frequency search. (a) Spectral spectrum shifted graph for thickness assumptions (set up four or five different layering systems). (b) Relationship between the thicknesses of the structure layer and the resonant frequency (red dot represents the thickness of asphalt surface layer).

As explained by Joh et al. [5], the frequency response curve consists of more than 400 frequencies to identify the resonant frequency and takes about 15 minutes to be analysed. Special algorithms have been created to analyse the frequency whereby it is divided into two analytical analyses of rough sweep and fine sweep. Then, four to five different layering systems have been set up and the results of this analysis form a graph of spectral displacements for the thickness assumptions whereby the blue line represents the frequency of rough sweeping and the red line represents the frequency of the fine sweeping (Figure 11(a)). The graph of the thickness and corresponding frequency is then plotted on a logarithmic scale and the relationship is found to be linear (Figure 11(b)). By using the resonant frequency (represented as a red dot) identified from the initial analysis, the thickness of the pavement layer structure can be determined.

### 5.4. Elastic Modulus of the Material

From the signal data obtained, the phase velocity dispersion curve can be derived through inverse analysis to produce the shear wave velocity profile. The elastic modulus of the asphalt surface layer material can be determined by using the value of this shear wave velocity using (2).  Table 2 shows the average value of the shear wave velocity and the average value of the elastic modulus of the material obtained in this study. Poisson's ratio has been estimated and the material mass density of the Poisson ratio used for the asphalt and aggregate mixture is 0.4 [15] and the unit weight of the material is 2200 kg/m^3^ [16]. The comparison between the theoretical dispersion curve and the experimental dispersion curve that produces the lowest root-mean square (RMS) error is used during the processing and analysis of data to obtain the shear wave values.

By using the average shear wave velocity data, the value of the elastic modulus for the asphalt surface layer can be determined. Within the range of temperatures that can occur in road pavements in Malaysia, elastic modulus values will vary from a few hundred MPa at high pavement temperatures to about 3000 MPa at the low end of pavement temperatures [15]. However, this study does not consider a low temperature environment because the temperature in Malaysia rarely falls below 30°C in daylight hours and is usually in the range of 35 to 45°C. From the calculation (Table 2), it was found that the range value of the elastic modulus is between 3929 MPa and 17726 MPa. The average elastic modulus obtained was higher than the standard set by the IKRAM Group Sdn. Bhd., which is 2500–3200 MPa. However, some of the elastic values are recorded within the Malaysian Public Work Department (PWD) standard (1200 MPa for wearing course and 1600 MPa for binder course) [15]. This might happen because the quality of the material at each location is different according to when the pavement was constructed.

From the measurements, high average elastic modulus values were recorded due to the influence of traffic load and pavement construction age [1]. The locations that have a high average elastic modulus, namely, Bunga Raya Road, Wira Road (Keris Mas College), Wira Road (UKM Transport Unit), Nik Ahmed Kamil Road, Tun Ismail Ali Road (gate number 3), and Faculty of Engineering's academic building, experience low traffic load while the Faculty of Engineering's administrative building is newly built. Rosyidi et al. [17] and Rosyidi [16] have studied the stiffness of new pavement and found that the average value of the elastic modulus on new roads is high. Therefore, it can be concluded that the elastic modulus value for new pavement is high compared to old pavement; a high elastic modulus represents the high strength of the pavement structure layers. However, the quality of pavement structure decreases with time and the rate of decrease depends on the traffic load, the quality of the materials, and environmental influences.

### 5.5. The Thickness of the Asphalt Surface Layer

The actual thickness of the asphalt surface layer is determined through the analysis of the wave propagation model, which requires a resonant frequency search. The thickness obtained was then compared to the design thickness specified by the Management Development Department, UKM (MDD UKM), and the Malaysian PWD standards.  Table 3 shows the comparison between the average asphalt surface layer thickness measured in this study and the design thickness of MDD UKM.

The research results reveal that the average thickness of the asphalt surface layer lies in the range between 0.04 and 0.08 m. Meanwhile, the thickness of the asphalt surface layer specified by Malaysian PWD is divided into two layers, namely, the wearing layer and the binder layer, and the range of each layer is 0.04 to 0.05 m and 0.04 to 0.1 m, respectively. Taking into account these two layers as the surface layer of asphalt, the range obtained is 0.04 to 0.14 m. The difference between the average thickness measured and the design thickness of MDD UKM is between 20 and 60%; the lowest difference of 26% was noted for Wira Road (UKM Transportation Unit) and the highest difference of 55.87% was recorded for Lebuh Ilmu Road. The average thickness of the asphalt surface layer is 0.04413 m. SASW and borehole studies done by Rosyidi [16] at Lebuh Ilmu Road revealed that the average thickness of the asphalt surface layer is 0.059 m and 0.0666 m, respectively.

Rosyidi [16] also conducted a study using the SASW and borehole method at Lebuh Ilmu Road. The results of this study show that the average thickness of the surface layer of asphalt is 0.059 m for the SASW method and 0.067 m for the borehole method. The difference in the thickness of the surface layer is due to the fact that the road was not properly constructed according to the standard and differences in the wave detected on the Gabor spectrum when IR filtration was carried out. This is because experience in analysing the spectrum of the wave data is needed to help the filtration process. Therefore, it can be concluded that the average thickness of the asphalt layer in this study is still within the allowable range of the standards set by Malaysian PWD and the design is almost similar to the design thickness of MDD UKM.

## 6. Conclusions

The following conclusions have been drawn from the study.

- The range of values of the elastic modulus of asphalt pavement materials obtained in this study, which are between 3929 MPa and 17726 MPa, is higher than the elastic modulus range set by the IKRAM Group Sdn. Bhd. However, some of the elastic values obtained are recorded within the Malaysian PWD standard. This happened because the quality of the material at each location is different according to when the pavement was constructed.
- The average thickness of the surface layer of asphalt as measured by the ERS method is between 0.04 and 0.08 m. This is still within the range of the standards set by Malaysian PWD, which is 0.04 to 0.14 m. Moreover, the difference between the thickness of the asphalt surface layer obtained in this research and the design thickness specified by the MDD UKM does not exceed 60%.
- The study shows that the ERS method using the PiScanner is able to measure the stiffness and thickness of the surface layer of asphalt according to the prescribed standards. Therefore, it can be concluded that this technique can be used not only for rigid pavements but also for determining the thickness and stiffness of flexible pavements.

## Figures and Tables

**Table 1:** The parameters used in the analysis.

Layer	Density	Poisson's ratio
1	2200	0.4
2	2130	0.35
3	1900	0.33

**Table 2:** Average of shear wave velocity and elastic modulus values.

Numbers	Location	Average of shear wave velocity values *V*_*S*_ (m/s)	Average of elastic modulus values *E* (MPa)
1	Gelanggang Road	1099.071	7705.032
2	Lebuh Ilmu Road	767.788	3928.877
3	Bunga Raya Road	1394.263	12263.678
4	Syed Nasir Road	1224.650	9634.834
5	Lingkungan Johan	1065.338	8020.023
6	Wira Road (Kolej Keris Mas)	1615.607	16999.285
7	Wira Road (UKM Transportation Unit)	1643.212	17726.012
8	Nik Ahmed Kamil Road	1460.126	13341.513
9	Temuan Road	897.858	5867.526
10	Tun Abdullah Mohd Salleh Hall	1173.139	8927.070
11	Tun Ismail Ali Road (gate number 3)	1567.664	15641.718
12	Tun Ismail Ali Road (Faculty of Law)	846.050	4863.557
13	Faculty of Engineering's administrative buildings	1266.959	14719.172
14	Faculty of Engineering's academic buildings	1263.280	13435.592
15	Faculty of Science and Technology's new buildings	1160.235	8643.595

**Table 3:** A comparison between the average asphalt layer thickness and the design proposed by MDD UKM.

Numbers	Location	Average asphalt layer thickness (m)	Design of MDD UKM (m)	Difference (%)
1	Gelanggang Road	0.05638	0.100	43.62
2	Lebuh Ilmu Road	0.04413		55.87
3	Bunga Raya Road	0.05681		43.19
4	Syed Nasir Road	0.05557		44.43
5	Lingkungan Johan	0.05020		49.80
6	Wira Road (Kolej Keris Mas)	0.04556		54.44
7	Wira Road (UKM Transportation Unit)	0.07400		26.00
8	Nik Ahmed Kamil Road	0.06409		35.91
9	Temuan Road	0.04480		55.20
10	Tun Abdullah Mohd Salleh Hall	0.05046		49.54
11	Tun Ismail Ali Road (gate number 3)	0.06062		39.38
12	Tun Ismail Ali Road (Faculty of Law)	0.05786		42.14
13	Faculty of Engineering's administrative buildings	0.04940		50.60
14	Faculty of Engineering's academic buildings	0.05467		45.33
15	Faculty of Science and Technology's new buildings	0.04700		53.00
